# Viviparous stingrays avoid contamination of the embryonic environment through faecal accumulation mechanisms

**DOI:** 10.1038/s41598-020-64271-2

**Published:** 2020-04-30

**Authors:** Taketeru Tomita, Masaru Nakamura, Yasuhisa Kobayashi, Atsushi Yoshinaka, Kiyomi Murakumo

**Affiliations:** 1 Zoological Laboratory, Okinawa Churashima Research Center, 888 Ishikawa, Motobu-cho, Okinawa, 905-0206 Japan; 2 Okinawa Chiraumi Aquarium, 424 Ishikawa, Motobu-cho, Okinawa, 905-0206 Japan; 3 Ushimado Marine Institute, Okayama University, 130-17, Ushimado, Okayama, 701-4303 Japan; 4 Noboribetsu Marine Park Nixe, 1-22, Noboribetsu-shi, Hokkaido, 059-0492 Japan

**Keywords:** Ichthyology, Embryology

## Abstract

In viviparous (live-bearing) animals, embryos face an embryo-specific defecation issue: faecal elimination *in utero* can cause fatal contamination of the embryonic environment. Our data from the viviparous red stingray (*Hemitrygon akajei*) reveals how viviparous elasmobranchs circumvent this issue. The exit of the embryonic intestine is maintained closed until close to birth, which allows the accumulation of faeces in the embryonic body. Faecal accumulation abilities are increased by (1) the large intestine size (represents about 400–600% of an adult intestine, proportionally), and (2) the modification in the intestinal inner wall structure, specialized to increase water uptake from the faecal matter. According to the literature, faecal accumulation may occur in embryos of the lamniform white shark as well. The reproductive biology of myliobatiform stingrays and lamniform sharks is characterized by the onset of oral feeding before birth (i.e. drinking of uterine milk and eating of sibling eggs, respectively), which is expected to result in the production of large amounts of faeces during gestation. The strong ability of faecal accumulation in these lineages is therefore likely an adaptation to their unique embryonic nutrition mechanism.

## Introduction

Viviparous vertebrates start oral feeding (i.e. drinking and eating) after birth. Some lineages of viviparous elasmobranchs, including myliobatiform stingrays (Batoidea, Myliobatiformes), provide a rare exception to this general view^1–3^. Similar to other elasmobranchs, during early gestation in myliobatiform stingrays, embryonic development relies on yolk absorption. However, during late gestational stages, yolk absorption is completed and the embryo further grows by drinking lipid-rich “milk” secreted from the uterine wall^4,5^. This nutrition mechanism, called lipid histotrophy, is so efficient that the embryo increases its weight with up to 1680–4900% during late gestation, which is among the greatest enlargements observed in viviparous elasmobranchs^6^.

One of the mysteries of lipid histotrophy concerns embryonic defecation. Generally, the embryo of viviparous animals faces an embryo-specific defecation issue: the passage of faeces *in utero* fatally contaminates the uterine environment^7^. This issue should be specifically critical in lipid histotrophy as (1) active digestion should produce a large amount of undigested materials and (2) the functional stomach and intestine frequently renew epithelial cells, both increasing the rate of faecal production during gestation. It is therefore likely that myliobatiform stingrays have some specific mechanism(s) to circumvent this issue, though this subject has never been investigated.

The present study reports for the first time faecal accumulation abilities in embryos of red stingray *Hemitrygon akajei* (Myliobatiformes, Dasiatidae), which is likely the mechanism allowing maintenance of a clean uterine environment. This species is commonly distributed around the main island of Japan, and its reproductive features were well described by ref. ^8^. However, knowledge of the embryonic development in this species is mostly restricted to external morphology^8^, whereas the physiological features of embryos remain largely unexplored. Thus, the aim of the present study was double: first, describe the ability of faecal accumulation in red stingray embryos, and then explore the morphological features that allow this ability.

## Results

### External morphology of the embryonic intestine of red stingray embryos

**Figure 1:**
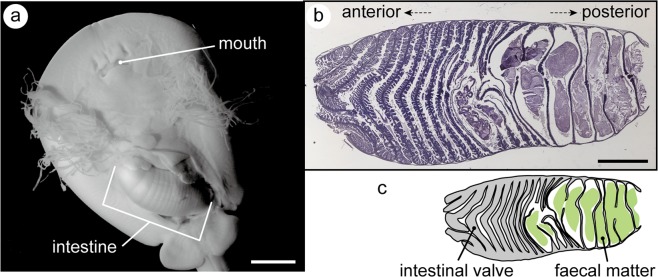
Embryonic intestine and faecal matter accumulation in the red stingray. (**a**) Ventral side of stage 32 embryo (the specimen is half-dissected to show the embryonic intestine). (**b**) Sagittal cross section through the intestine of a stage 32 embryo, and (**c**) its schematic representation. Note that the faecal matter only occupies the posterior half of the intestine. Scale bar = 500 µm.

Based on developmental staging criteria for batoid embryos by ref. ^9^, thick and viscous faecal matter was observed in the intestine of stage 32 and 33 red stingray embryos. The smallest embryo specimen that contained faecal matter weighed 2.16 mg (stage 32). The faecal matter occupied the posterior half of the intestine in stage 32 embryos (Fig. 1).

**Figure 2:**
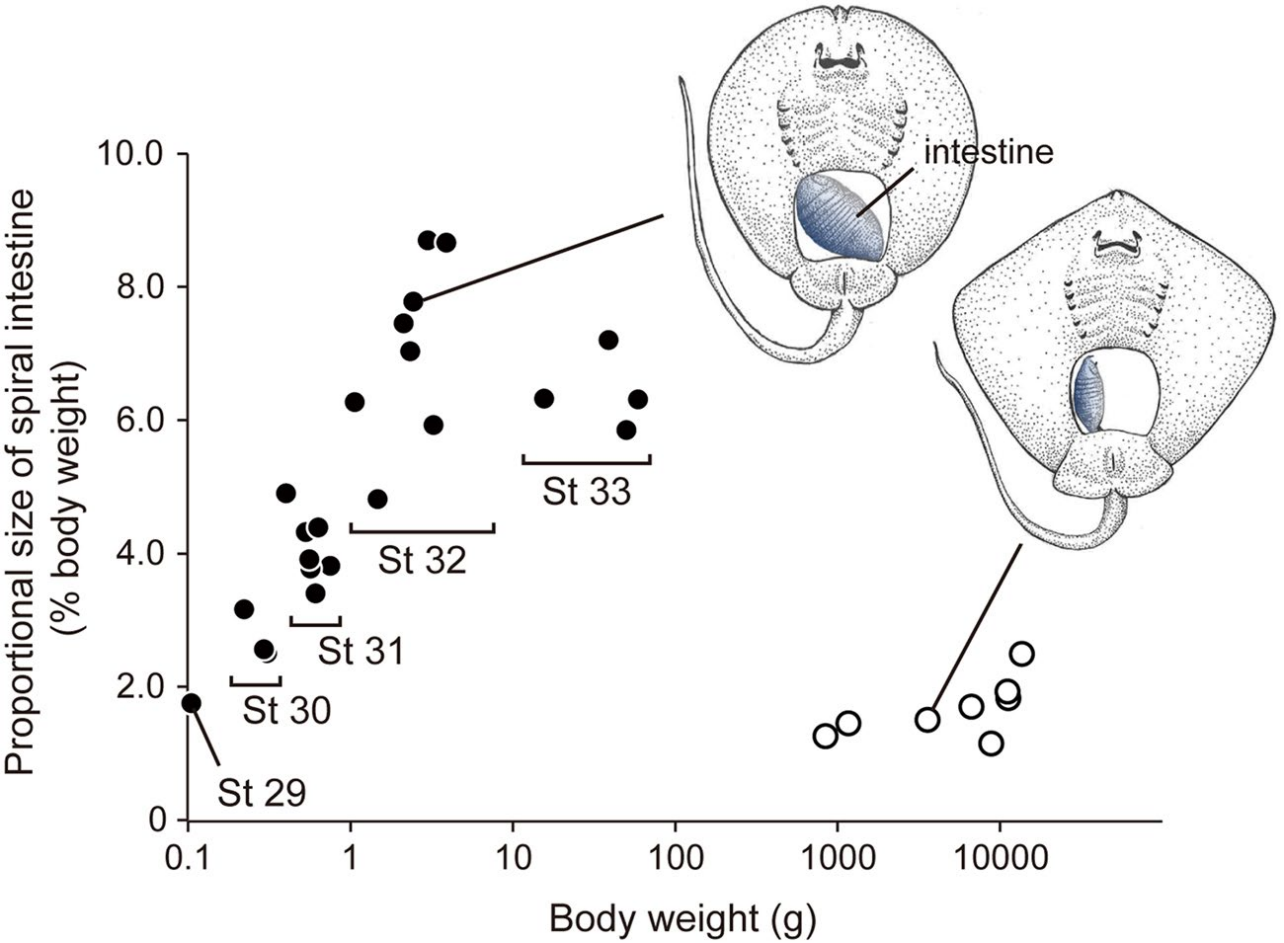
Changes in the proportional size (%body weight) of the intestine in red stingray embryos (black circles) and postnatal individuals (white circles). External gill filaments are not shown in the embryonic illustration.

The size of the intestine drastically changed with ontogeny (Fig. 2). In stage 29 embryos, the proportional size of the intestine (% body volume) was about 2.0%. This value increased to 6.0%–9.0% in stages 32 and 33, and dropped to the lowest level (1–2%) after birth. At stage 32, the posterior half of the intestine was filled with faecal matter, and the total faeces volume was calculated at about 3.0–4.5%.

## Histology of the embryonic intestine in red stingrays

**Figure 3:**
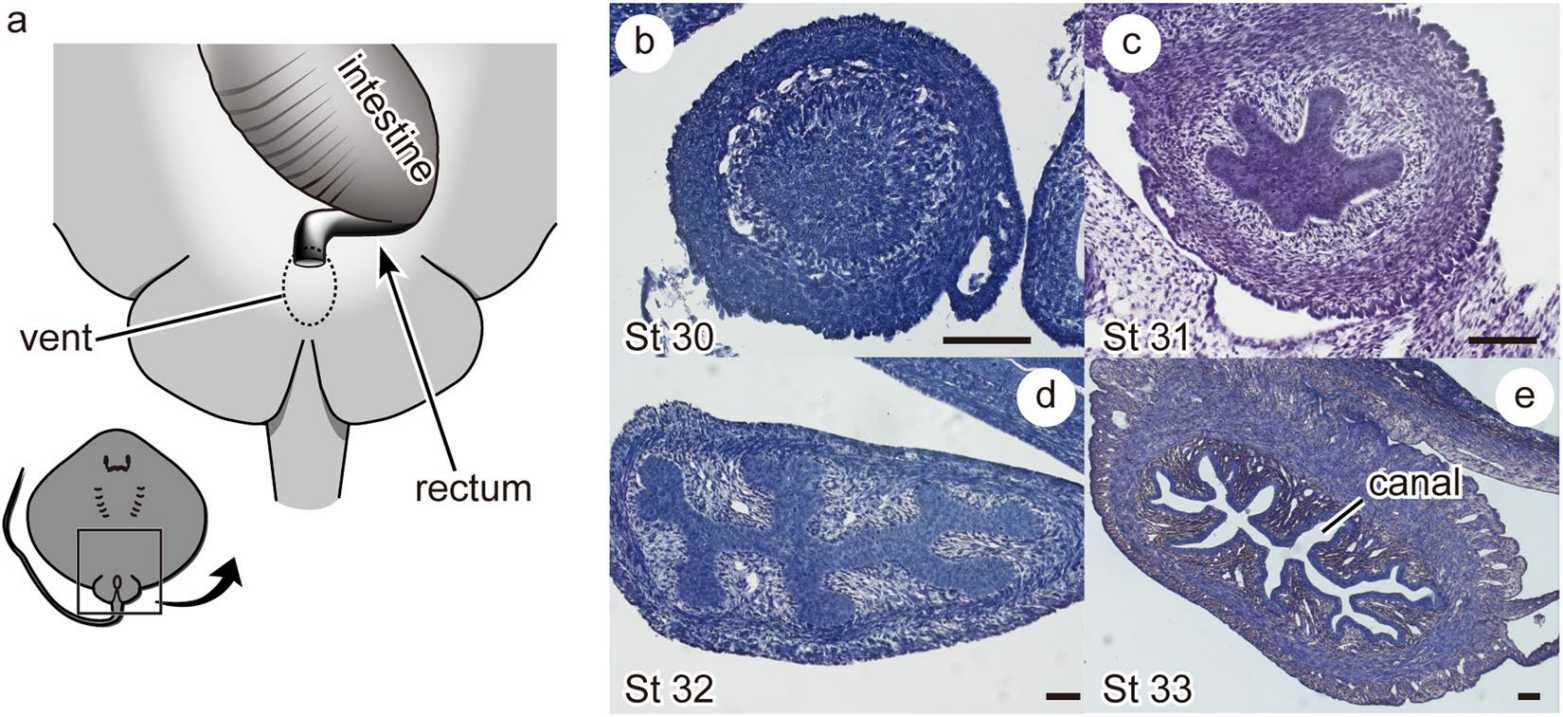
Morphological changes at the junction between the intestine and rectum during the embryonic development of the red stingray. (**a**) Schematic drawing of the distal part of the digestive tract. Transversal sections through the rectum show that the rectal canal is not fully developed and closed in stage 30-32 embryos (**b**-**d**), but is opened in stage 33 embryos (the latest stage) (**e**). Scale bar = 100 µm.

Histological sections showed that the exit of the intestine remained closed until close to birth (Fig. 3): Cross sections cut perpendicular to the rectum showed that the rectal canal is not fully formed at the junction between the rectum and intestine; thus, the rectal canal is not connected to the intestine from embryo stage 29 (earliest-observed stage) through stage 32 (Fig. 3b-d). Formation of the rectal canal is completed during the latest embryonic stage (stage 33, Fig. 3e).

**Figure 4:**
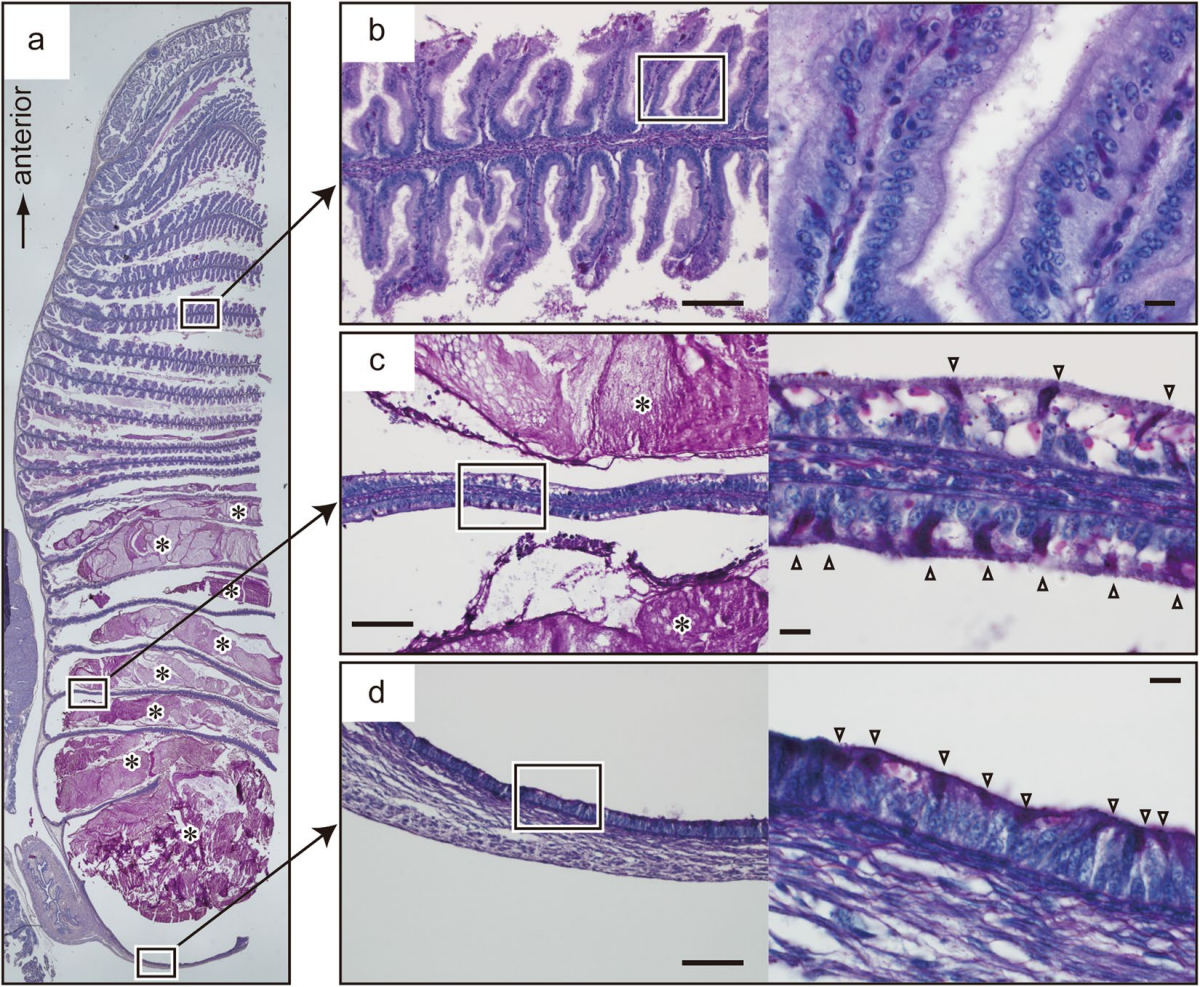
(**a**) Periodic acid-Schiff (PAS)-stained histological sections through the intestine of stage 32 red stingray embryos. Faecal matter (*) accumulated in the posterior half of the intestine. (**b**) The fifth row of spiral valve (left) and its close-up view (right). (**c**) The 17th row of the spiral valve (left) and its close-up view (right). (**d**) The posterior end of the intestine and its close-up view. Densely distributed PAS-positive goblet cells (white triangles) are seen in the right panels in c and d. Scale bars =100 µm in left panels of **b**-**d**; 10 µm in right panels of **b**-**d**.

The anterior half-rows of the intestinal valves (anterior 9–10 out of 20 rows) were covered with well-developed villi (Fig. 4a,b). The surface of villi consisted of tall absorptive epithelial cells, with the apical surface covered by numerous microvilli (Fig. 4b). In contrast, the posterior 10–11 rows of intestinal valves were thin and lacked villi (Fig. 4a,c). Goblet cells were densely distributed on the inner surface of the posterior portion of the intestinal wall (Fig. 4c,d).

**Figure 5:**
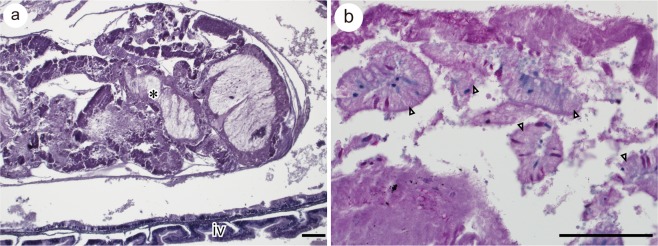
(**a**) Internal structure of faecal matter (*) in stage 32 red stingray embryos showing its heterogeneous features. (**b**) Close-up view of PAS-stained faecal matter. Fragmented intestinal epithelia (white triangles) are seen. Scale bars =100 µm.

The internal structure of faecal matter is heterogeneous, composed of several types of materials with different degrees of reaction to hematoxylin and eosin or periodic acid–Schiff (PAS) stain (Fig. 5a). Fragmented intestinal epithelium is also included (Fig. 5b). These features suggest that faecal matter is a mixture of several materials of different origins, such as nutrients, dead epithelia, and digestive secretions, though this could not be confirmed in the present study.

## Comparison with oviparous species (*Raja pulchra*)

**Figure 6:**
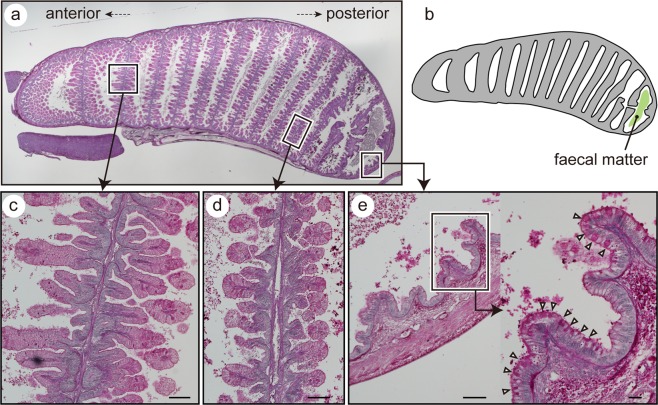
(**a**) PAS-stained histological section through the intestine of stage 32 embryos of oviparous batoid (*Raja pulchra*), and (**b**) its schematic representation. Note that faecal matter is restricted to the most posterior region of the intestine. (**c**) The second row of spiral valve, and (**d**) the 10th row of the spiral valve. (**e**) The posterior end of the intestine (left) and its close-up view (right). Densely populated PAS-positive goblet cells (white triangles) are seen in the right panel in **e**. Scale bars =100 µm in **c**, **d** and left panel of **e**; 10 µm in right panel of **e**.

The average (± standard deviation) of relative intestine size (% body weight, yolk-sac removed) of stage 32 and 33 embryos were 1.05% (±0.29, n = 3) and 1.56% (±0.34, n = 6), respectively. There were no significant differences between the relative intestinal sizes of the embryos at stages 32 and 33 and those of postnatal individuals (1.59% [±0.25, n = 4];>0.05, t-test). The entire structure was similar to that observed in the red stingray, but presented several distinguishing features. Firstly, the amount of faecal matters was smaller, only occupying the most posterior region of the intestine (Fig. 6a,b). Secondly, the intestinal valves were covered with well-developed villi throughout almost the whole length of the intestine (Fig. 6c,d). Thirdly, the inner wall at the posterior-end of the intestine was also covered with villi. These villi were shorter than those covering the intestinal valves, and goblet cells were densely distributed on its surface (Fig. 6e).

## Discussion

The red stingray embryo accumulates all embryonic faeces until birth, which could be the mechanism to maintain clean the uterine environment. This phenomenon is supported by three lines of evidence: (1) the rectal canal is kept closed at the junction between the intestine and rectum until close to birth, which prevents the passage of faeces through the rectum; (2) the embryonic intestine is largely filled with faecal matters; and (3) the newborn juveniles of some myliobatiform stingrays, *Himantura uarnak* and *Urogymnus asperrimus*, were observed to eliminate faeces before the first food intake after birth (K.M., unpublished data based on five observations at Okinawa Churaumi Aquarium between 2016–2019). A similar phenomenon was well known in the mammalian fetus (i.e. meconium storage^10^), but has been less investigated in other viviparous vertebrates. To the best of our knowledge, the only previous record of embryonic faecal accumulation in elasmobranchs, other than myliobatiform stingrays, came from ref. ^11^. They reported that a large amount of “greenish-brown material” was present in the intestine of embryonic white shark *Carcharodon carcharias* (Lamniformes, Lamnidae), though the physiological implications of this phenomenon were not discussed. Regarding viviparous fishes other than Elasmobranchii, the Goodeidae, a well-known viviparous teleost, the embryo of which has ribbon-like structure extended from the perianal region to absorb nutrients from the uterine fluid, may lack the ability for faecal accumulation; this is because it presents unclosed termini of embryonic intestine and no accumulated faecal matter in the intestine (Figs. 3 and 4 in ref. ^12^; Fig. S1 in ref. ^13^).

The restricted taxonomic distribution of embryonic faecal accumulation among elasmobranch species raises the hypothesis that this ability evolved in association with the reproductive strategy. The reproductive biology of elasmobranchs exhibits high diversity, especially concerning the mechanisms of embryo nutrition^6^. Generally, the embryonic nutrition of myliobatiform stingrays and white sharks is categorized into different modes: lipid histotrophy and oophagy, respectively (e.g., ref. ^6^). The former mode, embryonic development is accomplished by drinking uterine “milk”, whereas in the latter mode by eating sibling eggs. Despite these differences in embryonic nutrition, these reproductive modes present a great similarity as embryos start oral feeding before birth. This mechanism requires embryos to have an active digestive process^4^ and may lead to the production of large amounts of faeces during gestation. Therefore, it is very likely that embryonic faecal accumulation observed in stingrays and probably in white sharks is a mechanism to maintain clean the uterine environment, considering their embryonic nutrition mechanism.

The unique origin of embryonic faecal matter in these elasmobranch taxa may reflect the composition of embryonic faecal matter. The composition of faecal matter has been well explored in mammals (humans); it comprises a large content of water, epithelial cells from the gastrointestinal tract and skin, hair, fatty material from the vernix caseosa, and bile acids (full description found in ref. ^14^). Considering the mammalian fetus acquires nutrients through the placenta without an intestinal digestive process, the composition of faecal matter in stingrays and lamniform sharks should be different from that in mammals, possibly including large proportions of undigested remains and digestive secretions. This hypothesis can be tested by measuring the chemical and protein composition of faecal material, although this task is beyond the scope of this study.

We found several morphological modifications that increase the faecal accumulation abilities in red stingray embryos. The first modification is the large intestine size, about 400–600% of the postnatal individual in relative size. This feature may contribute to provide enough space to accumulate faeces. Another modification is the large proportion of the “colon-like” region in the embryonic intestine. A previous study showed that the posterior-end region of the batoid intestine is distinct from the other region as it lacks nutrient-absorptive villi and presents densely distributed goblet cells^15^. This region was found to have strong water absorptive ability, thus was considered as a functional analogue of the mammalian colon^15,16^. Intestinal goblet cells may have multiple functions, represented by the production of mucus for coating of the faecal surface, as known in the mammalian colon. However, large amounts of aquaporin-4, a water channel protein in mammals, is produced in goblet cells in the batoid intestine^15^. Thus, highly populated goblet cells in colon-like regions may also be responsible for their strong water absorption abilities. Our observations showed that the proportion of this colon-like region is approximately 10 times greater in the embryonic intestine than in that of postnatal individuals (posteriorly, the colon-like region occupied ~50% of the intestinal length in embryos and ~5% in postnatal individuals), suggesting stronger abilities of water uptake from faeces in embryos. These features contrast sharply with those of oviparous batoids: intestine size in oviparous *Raja pulchra* remains unaltered in pre- and post-hatching individuals. In addition, in oviparous species such as *Leucoraja erinacea* and *Raja pulchra*, the colon-like region in the embryonic intestine is, similar to that in the adult intestine, restricted only to a small part toward the posterior end^16^.

To summarize, the present study provides novel evidences that support embryonic faecal accumulation in viviparous elasmobranchs. It has been assumed that the uterine environment is controlled by the mother in viviparous elasmobranchs. Therefore, many studies have focused on uterine mechanisms to achieve this purpose, such as ammonia detoxification^17^, respiratory-gas exchange^4,18^, and periodic replacement of the uterine fluid with external seawater (uterine flushing)^19,20^. Faecal accumulation in embryonic myliobatiform stingray presented in this study showed that the embryo also plays an essential role in controlling the uterine environment.

## Materials and Methods

### Collection of embryo specimens

A total of 24 embryo specimens were collected from pregnant red stingrays, accidently caught by local fishermen at Seto Inland Sea and the eastern coast of Kyushu Island, Japan, and donated to the Ushimado Marine Institute in Okayama University (Okayama, Japan) and the University of Miyazaki (Miyazaki, Japan), respectively. Maintenance and handling of the fish were conducted in strict accordance with the Okayama University Guide for Care and Use of Laboratory Animals (http://www.cc.okayama-u.ac.jp/∼animal/committee.html). We performed procedures on the fish with the same consideration given to higher vertebrates (i.e. reptiles, birds, and mammals). However, approval from the Okayama University Animal Care and Use Committee was not obtained because although such approval is mandatory for the use of higher vertebrates, the Guide waives that requirement for protocols involving the use of lower vertebrates (i.e. fish and amphibians). All methods were performed in accordance with the relevant guidelines and regulations. Embryos ranged between 0.11 and 59.0 g in body weight (BW) and were between stages 29 and 33. Developmental staging of embryo specimens was performed according to that described in a previous report on the oviparous skate *Leucoraja ocellata*^9^.

### External morphology

External morphology of the spiral intestine was observed using a digital microscope (Keyence VHX-1000; Keyence Co.) at Okinawa Churashima Research Center (Okinawa, Japan). The volume of the intestine was calculated as 1/6 π × (intestinal length) × (intestinal width)^2^, assuming the spheroid morphology. For calculating the proportional volume of the intestine, body weight is converted into body volume, assuming a body density of 1.0 g/cm^3^. Body weight was measured in fixed specimens using an analytical balance (GR-120; A&D Company Limited). Statistical analysis (t-test) was performed using the built-in statistical tool in Excel 2019 (Microsoft Co.).

### Histology

Embryonic intestines were fixed in Bouin’s solution for 2–3 days. Then, they were dehydrated with a series of ethanol and xylene solutions, and embedded in paraffin. Paraffin-embedded samples were cut into 7 µm sections. Following deparaffinization with xylene, they were rehydrated in graded alcohol solutions, and stained with hematoxylin and eosin or periodic acid-Schiff (PAS) stain, for detection of mucosubstance. Goblet cells were identified based on their characteristic morphology and positive reaction with PAS staining^16^.

### Comparison with oviparous species

*Raja pulchra* eggs were collected from a single captive specimen at Noboribetsu Marine Park Nixe (Hokkaido, Japan) in 2018, and were incubated at the Marine Research Center of University of the Ryukyus (Okinawa, Japan) at a water temperature of 11°C. A total of 8 embryos were euthanized with phenoxyethanol, and then fixed in Bouin’s solution. Embryos ranged between 13.3 and 23.6 g in BW (excluding the yolk weight), and from stage 32 to 33 in developmental stage. Morphological and histological observations were conducted in the same way as those for red stingrays, described above. For comparison, we also observed 4 postnatal specimens (19.3 to 29.2 g in BW).
